# Cost analysis of smear microscopy and the Xpert assay for tuberculosis diagnosis: average turnaround time

**DOI:** 10.1590/0037-8682-0314-2020

**Published:** 2020-09-25

**Authors:** Lida Jouca de Assis Figueredo, Silvana Spíndola de Miranda, Lucas Benício dos Santos, Caroline Gontijo Gonçalves Manso, Valéria Martins Soares, Suely Alves, Maria Cláudia Vater, Afrânio Lineu Kritski, Wânia da Silva Carvalho, Cristiane Menezes de Pádua, Isabela Neves de Almeida

**Affiliations:** 1 Universidade Federal de Minas Gerais, Laboratório de Pesquisa em Micobactérias, Belo Horizonte, MG, Brasil.; 2 Universidade Federal de Minas Gerais, Grupo de Pesquisa em Micobacterioses, Faculdade de Medicina, Belo Horizonte, MG, Brasil.; 3 Fundação Hospitalar do Estado de Minas Gerais, Hospital Júlia Kubistchek, Laboratório de Microbiologia, Belo Horizonte, MG, Brasil.; 4 Universidade Federal do Rio de Janeiro, Programa Acadêmico de Tuberculose, Rio de Janeiro, RJ, Brasil.; 5 Universidade Federal de Minas Gerais, Faculdade de Farmácia, Belo Horizonte, MG, Brasil.

**Keywords:** Mycobacterium tuberculosis, Molecular diagnostic, Pulmonary tuberculosis, Cost analysis, Health system

## Abstract

**INTRODUCTION:**

Rapid and accurate tuberculosis detection is critical for improving patient diagnosis and decreasing tuberculosis transmission. Molecular assays can significantly increase laboratory costs; therefore, the average time and economic impact should be evaluated before implementing a new technology. The aim of this study was to evaluate the cost and average turnaround time of smear microscopy and Xpert assay at a university hospital.

**METHODS:**

The turnaround time and cost of the laboratory diagnosis of tuberculosis were calculated based on the mean cost and activity based costing (ABC).

**RESULTS:**

The average turnaround time for smear microscopy was 16.6 hours while that for Xpert was 24.1 hours. The Xpert had a mean cost of USD 17.37 with an ABC of USD 10.86, while smear microscopy had a mean cost of USD 13.31 with an ABC of USD 6.01. The sensitivity of smear microscopy was 42.9% and its specificity was 99.1%, while the Xpert assay had a sensitivity of 100% and a specificity of 96.7%.

**CONCLUSIONS:**

The Xpert assay has high accuracy; however, the turnaround time and cost of smear microscopy were lower than those of Xpert.

## INTRODUCTION

Tuberculosis (TB) has existed for millennia and remains a major global health problem^1^. The World Health Organization (WHO) estimated that in 2018, approximately 10 million people had developed TB, and that TB was responsible for an estimated 1.2 million deaths among human immunodeficiency virus (HIV) negative patients and an additional 251 thousand deaths among patients living with HIV^1^.

Brazil is one of 30 countries with a high TB burden, accounting for 84% of the global total number of cases^1^. In 2018, 78,652 new cases were registered, and only 34% of them were tested using the Xpert^®^ MTB/RIF assay (Cepheid, Sunnyvale, CA, USA) (Xpert). Moreover, 79% of the new patients knew their HIV status, and 87% of the identified cases were pulmonary TB^1^.

TB typically affects the lungs, but can also spread to other sites^1^. The diagnosis of extrapulmonary TB remains a challenge, since the total number of bacteria in extrapulmonary specimens is often lower than that present in pulmonary specimens^2^. Furthermore, the collection of extrapulmonary material requires invasive procedures, and it is usually difficult to obtain additional samples^2^.

Accurate, rapid detection of TB is critical for improving patient care and decreasing TB transmission^1^. The Xpert assay is an automated molecular test that can detect both TB and rifampicin resistance, generally within two hours after starting the test, with minimal hands-on technical time^3^.

The WHO issued initial recommendations concerning the Xpert test in early 2011^4^. In a systematic review, its sensitivity in detecting TB in pulmonary samples ranged from 58% to 100%, whereas the specificity ranged from 86% to 100%^3^.

The authors highlighted that the Xpert test is expensive, and that further research is needed to evaluate its use in TB programs and to assess if this investment could help start treatment promptly and improve health outcomes^3^. Despite the WHO recommendations for the use of the Xpert system, sputum smear microscopy remains widely used in many countries for the rapid diagnosis of TB under routine conditions. This method has a variable sensitivity ranging from 32% to 89% and a specificity between 85% and 100%^4^ ^,^ ^5^. 

TB control programs must balance costs with performance characteristics and the need for rapid results^1^ ^,^ ^3^ ^,^ ^6^. Intensive implementation of molecular assays may lead to significant increases in laboratory cost. Selective implementation of molecular assays could be considered for some settings^6^, and accurate and rapid detection of TB is critical for improving patient outcomes (increased cure and decreased mortality rates, additional drug resistance, treatment failure, and relapse) and decreasing TB transmission^3^.

Thus, the aim of this study was to evaluate and compare the costs and average time to completion of smear microscopy and Xpert assay at a teaching hospital.

## METHODS

### Study design, variables and study site

The present study assessed respiratory and non-respiratory specimens (n=1009) that had been received at the Research Laboratory in Mycobacteria of the School of Medicine of the Federal University of Minas Gerais (UFMG), between November 2014 and November 2015. Of these, 141 patients accepted to participate, signed the term of free informed consent, and were included in the study of the accuracy and time of laboratory tests and in the analysis of clinical and sociodemographic characteristics.

Clinical and sociodemographic data were obtained using a standardized questionnaire and a review of the patients’ records on their behavior (alcohol and tobacco use), HIV infection, and any abnormalities on pulmonary imaging (chest x-ray or computed tomography). A pattern suggestive of TB was the presence of cavitation, infiltrate in upper lobe and apical segment of the lower lobe or mediastinal enlargement, or increased hilar lymph node or miliary pattern or pleural effusion or confluent parenchymal opacities, confluent parenchymal opacities, characterized as a budding tree^7^. 

These patients received medical care at the teaching hospital of the UFMG, a public and general university hospital that conducts educational, research, and medical care activities. This institution consists of one hospital unit and seven outpatient care centers^8^ ^,^ ^9^. Direct sputum smear examinations that were urgently requested were excluded from clinical specimens, since at the hospital, the Xpert system is not used immediately.

### Processing of clinical samples

The specimens were processed following the standard *N-acetyl-1-cysteine* and sodium hydroxide (NALC/NaOH) method with a final NaOH concentration of 1%^10^. After this step, the sediment was resuspended in 1.0 to 1.5 mL of sterile water and used for smear microscopy, Xpert testing, and culture tests. 

*Smear microscopy:* All smears prepared from decontaminated samples were stained with auramine O and analyzed by fluorescence microscopy^10^. 

*Xpert* ^*®*^ *MTB/RIF*: This assay was performed according to the manufacturer’s instructions (Cepheid, Sunnyvale, CA, USA). Briefly, a sample reagent was added in a 3:1 ratio to ≥0.5 mL of decontaminated specimen. The closed tube was agitated manually twice during a 15-min incubation at room temperature. Then, 2 mL of the inactivated sample reagent-sample mixture was transferred to the Xpert test cartridge. The cartridges were inserted into the Xpert device, and the automatically generated results were read after 90 min^3^ ^,^ ^11^. 

### Average time: smear microscopy and Xpert

To calculate the average time to the release of test results, we took into consideration the hour when the samples were received in the collection sector and the hour when test results were released in the system. This period includes the times of receipt of samples, internal registration, the process of decontamination/centrifugation of the samples, separation of the aliquots for each diagnostic test, execution of the tests, release of results by the technician, and release of the results into the computerized system of the hospital.

The times during which tests were performed urgently via direct smear, at night, on weekends and holidays were not considered; only those performed routinely with NALC/NaOH were considered.

### Cost analysis

The cost analysis of the TB laboratory diagnosis was based on the mean cost and activity based costing (ABC). The mean cost was calculated by dividing the total costs by the quantity produced over a determined period of time^12^, which considered the total number of examinations performed in a month. The ABC principle is suitable for complex organizations, such as hospitals, where products use consumer resources in a highly heterogeneous manner^9^ ^,^ ^12^, therefore covering as many direct and indirect costs as possible through cost drivers. The component cost evaluation and calculations were performed as described by Almeida et al. 2017 and the costs were expressed in USD, using the conversion rate of USD 1.00 = R$ 4.03, as established by the Central Bank of Brazil in 2019 (https://www.bcb.gov.br/)^9^ ^,^ ^13^ ^,^ ^14^. 

### Statistical analysis

Descriptive analysis was performed to characterize the study population concerning the selected variables. Measures of central tendency and dispersion were used for continuous variables, and absolute and relative frequencies for categorical variables. 

The sensitivity and specificity of *Xpert* ^*®*^ *MTB/RIF* and smear microscopy with their 95% confidence intervals (CIs) were estimated, comparing the results to those of the phenotypic identification test as a standard method^10^. All analyses were performed using SAS version 9.4 (SAS Institute Inc., Cary, NC, USA).

### Ethical considerations

The study protocol was approved by the Research Ethics Committee of the UFMG (protocol numbers CAAE-11821913.6.000.5257 and CAAE 0223.2412.7.1001.5149, and DEPE/HC protocol number 139/12).

## RESULTS

**FIGURE 1:**
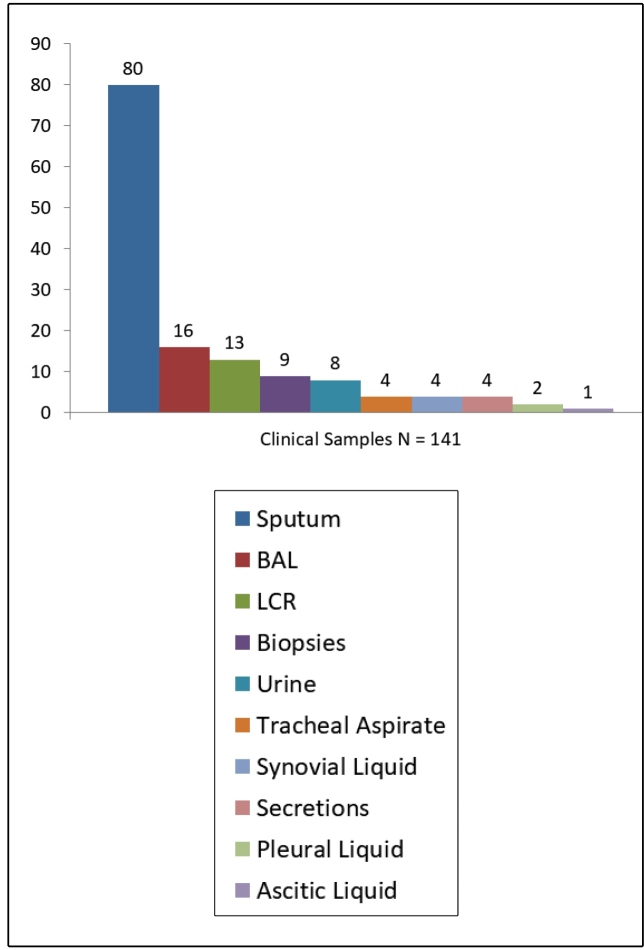
The distribution of clinical samples.

The study included 141 patients’ clinical samples: 100 pulmonary specimens and 41 extrapulmonary specimens (Figure 1); 66% (93/141) of the patients were inpatients and 34% (48/141) were outpatients. 

The sociodemographic characteristics of these patients are presented in Table 1. Most patients were male, aged 46 to 60 years (median=46.0), single, had non-white skin, had nine or fewer years of formal education, and 25 had pulmonary TB. Between 20% and 40% of the patients were reported to be current users of alcohol and tobacco, respectively. Regarding radiological patterns, 27% presented presumed TB abnormalities. 

**TABLE 1:** Distribution of sociodemographic characteristics (n=141)*

	Frequency	
Variables	Total number of patients	
	n	%
Sociodemographic		
Age, years		
18-35	39	28.7
36-45	27	19.9
46-60	37	27.2
61-88	33	24.3
Gender		
Male	83	58.9
Skin color		
Non-white	84	80.0
White	21	20.0
Education, years		
Illiterate	5	8.2
≤ 9	39	63.9
≥10	17	27.9
Marital status		
Married	57	48.3
Single	61	51.7
Behavioral		
Alcohol use (CAGE)		
Positive	11	40.7
Negative	16	59.3
Tobacco use		
Never	30	35.3
Current	18	21.2
Ever	37	45.5
Clinical		
Prescribed TB therapy		
RHZE	24	17.0
Special regimen	1	0.7
None	116	82.3
HIV status		
Positive	26	13.5
Negative	37	26.2
Unknown result/not tested	78	55.3
Radiological patterns		
Suggestive of TB	38	27.0
Without abnormalities	7	5.0
Other abnormalities	37	26.2
Not performed	59	41.8

**CAGE** alcohol questionnaire; **TB:** tuberculosis; **RHZE:** rifampicin, isoniazid, pyrazinamide, ethambutol. *missing values were excluded.

Accuracy assessment of the smear microscopy revealed a sensitivity of 42.9% (95% CI, 24.5-61.2) and a specificity of 99.1% (95% CI, 97.2-100.0). The accuracy assessment of the Xpert assay revealed a sensitivity of 100% (95% CI, 1.00-1.00) and a specificity of 96.7% (95% CI, 93.53-99.88).

Positive Xpert assays and negative culture test results were observed for the samples of two patients in retreatment, while 11 positive culture test results for nontuberculous mycobacteria (NTM) were not detected by the Xpert system. 

The average time to smear microscopy results was 16.6 hours and the median time was 9.3 hours (standard deviation, 15.1 hours; range, 1.2-48 hours). The mean time to completion of the Xpert assay was 24.1 hours, with a median time of 24 hours (standard deviation, 18.2 hours; range, 2-72 hours). 

### Cost analysis

**FIGURE 2:**
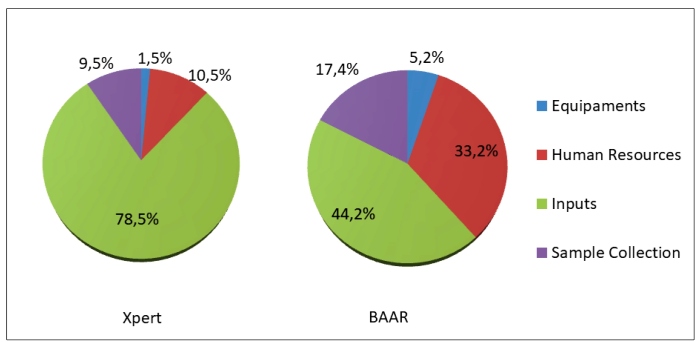
The impact of ABC components.

The mean cost of the Xpert assay was USD 17.37 with an ABC value of USD 10.86, while the mean cost of the smear microscopy was USD 13.31 with an ABC value of USD 6.01. Proportionally, the impact of cost components of smear microscopy was lower than that of Xpert, except for the human resources component. The component cost that impacted the mean costs and ABC values of Xpert and smear microscopy are described in Figure 2.

## DISCUSSION

The study revealed that the average time to completion of the Xpert assay result was longer than that of sputum smear microscopy. This is because the slide is read immediately, but the sample undergoes processing in the Xpert system. In addition, the fact that in our laboratory we use the equipment with only four modules might also play a role. Nevertheless, although smear microscopy is cheaper than the Xpert assay, the latter is more accurate.

The advantage of the Xpert system is that it offers the possibility for a fully automated test after insertion of the clinical specimen into the cartridge. Assay steps (DNA extraction, amplification, and detection) are independent of manual technical interventions, thus minimizing analytical errors and improving quality control^3^. 

The sensitivity and specificity values found in this study (100% and 96.7%, respectively) are similar to the results of a meta-analysis that included 27 studies with a total of 9500 specimens analyzed using the Xpert system, and which presented a range of sensitivity between 58% and 100% and specificity between 86% and 100%^3^. 

The specificity was found to be lower than the sensitivity due to the presence of *M. tuberculosis* complex DNA in two cases of treatment control patients who presented negative culture tests. Therefore, the recommendation not to use the Xpert system for treatment control should be reinforced, except for probable TB in new cases, if resistance to rifampicin or multidrug resistance is suspected.

In this study, the Xpert system did not show false positive results among NTM isolates, mainly due to the increase in prevalence of disease-associated NTM, as has already been described by other authors^15^ ^-^ ^17^. Therefore, NTM should be suspected in conditions where smear microscopy tests yield positive results and are undetected by the Xpert assay^18^. Our data agree with the results of a meta-analysis that evaluated 14 studies including 180 NTM cases and showed that the false positivity of the Xpert assay for these pathogens was only 0.6%^3^, as well as another study that demonstrated a specificity of 97.8%^19^. 

The average times extrapolated from the results (16.3 and 24 hours for smear microscopy and Xpert assay, respectively) are due to the fact that the laboratory does not work at night, on weekends, and holidays with processing of samples with NALC/NaOH; thus, the clinical samples are stored for the test to be carried out on the next business day. The recommendation of the Ministry of Health and reinforced by other authors is that routine results should be released within 24 hours^11^ ^,^ ^18^ ^,^ ^20^ ^,^ ^21^, which was seen from the mean of our results.

The ABC value of the Xpert assay was higher than that of smear microscopy and the input was the component with the greatest impact. However, it should be noted that the Xpert system is subsidized in Brazil^22^. Efforts are being made to increase the coverage area of the Xpert system worldwide through subsidies to make it available in developing and underdeveloped countries, where health systems work under heavy economic constraints^22^ ^,^ ^23^. 

This is mainly due to the demand of exclusive inputs from the supplier that are imported and undergo exchange variation, which is a dynamic and sensitive variable to the economic scenario of each country^24^.

When incorporating new technology and comparing it with already existing technology, it is important to evaluate all operational and technical aspects. Managers need to be aware of the relevant outcomes of TB treatment and bacillus transmission in the community when evaluating the advantages and disadvantages of incorporating new diagnostic tests^25^ ^,^ ^26^. 

The results described in this paper should alert Brazilian researchers to the need for the development and validation of a national molecular test. Although the Xpert system has high accuracy in the diagnosis of TB, national technological independence is necessary to ensure that cost is not a variable that hinders or even impedes the incorporation of a tool that is effective for the rapid and accurate diagnosis of TB^22^ ^,^ ^27^. 

The results should not be extrapolated to other scenarios owing to differences in the components analyzed. Thus, each site must perform its own analysis.

In conclusion, the Xpert assay has high accuracy; however, the time to sputum smear test results and the ABC value were lower than those observed for the Xpert system.
